# Nanolayers of Poly(*N*,*N′*-Dimethylaminoethyl Methacrylate) with a Star Topology and Their Antibacterial Activity

**DOI:** 10.3390/polym12010230

**Published:** 2020-01-17

**Authors:** Paulina Teper, Joanna Chojniak-Gronek, Anna Hercog, Natalia Oleszko-Torbus, Grażyna Płaza, Jerzy Kubacki, Katarzyna Balin, Agnieszka Kowalczuk, Barbara Mendrek

**Affiliations:** 1 Centre of Polymer and Carbon Materials, Polish Academy of Sciences, M. Curie-Sklodowskiej 34, 41-819 Zabrze, Poland; pteper@cmpw-pan.edu.pl (P.T.); ahercog@cmpw-pan.edu.pl (A.H.); noleszko@cmpw-pan.edu.pl (N.O.-T.); akowalczuk@cmpw-pan.edu.pl (A.K.); 2 Institute for Ecology of Industrial Areas, Kossutha 6, 40-844 Katowice, Poland; j.chojniak-gronek@ietu.pl (J.C.-G.); g.plaza@ietu.pl (G.P.); 3 A. Chelkowski Institute of Physics, University of Silesia, Uniwersytecka 4, 40-007 Katowice, Poland; jerzy.kubacki@us.edu.pl (J.K.); katarzyna.balin@us.edu.pl (K.B.); 4 Silesian Center for Education and Interdisciplinary Research, 75 Pulku Piechoty 1A, 41-500 Chorzów, Poland

**Keywords:** star polymers, antibacterial surfaces, poly(*N*,*N*′-dimethylaminoethyl methacrylate), “grafting to” method, antibacterial activity

## Abstract

In this paper, we focus on the synthesis and characterization of novel stable nanolayers made of star methacrylate polymers. The effect of nanolayer modification on its antibacterial properties was also studied. A covalent immobilization of star poly(*N*,*N*′-dimethylaminoethyl methacrylate) (PDMAEMA) to benzophenone functionalized glass or silicon supports was carried out via a “grafting to” approach using UV irradiation. To date, star polymer UV immobilization has never been used for this purpose. The thickness of the resulting nanolayers increased from 30 to 120 nm with the molar mass of the immobilized stars. The successful bonding of star PDMAEMA to the supports was confirmed by surface sensitive quantitative spectroscopic methods. Next, amino groups in the polymer layer were quaternized with bromoethane, and the influence of this modification on the antibacterial properties of the obtained materials was analyzed using a selected reference strain of bacteria. The resulting star nanolayer surfaces exhibited higher antimicrobial activity against *Bacillus subtilis* ATCC 6633 compared to that of the linear PDMAEMA analogues grafted onto a support. These promising results and the knowledge about the influence of the topology and modification of PDMAEMA layers on their properties may help in searching for new materials for antimicrobial applications in medicine.

## 1. Introduction

In hospitals and pharmacies, along with the food and cosmetology industries, it is important to maintain the sterility of materials in terms of human health and life. Therefore, in recent years, interest in surfaces with antibacterial properties has significantly increased. Such materials stop the growth of bacteria, viruses, or fungi and simultaneously enable the destruction of microorganisms.

In addition, a reduced antibiotic activity, due to an increase of bacterial resistance, is one of the biggest problems facing medicine, and it is important to find new solutions to this issue.

Therefore, to eliminate or reduce bacterial attachment and biofilm formation on various supports, e.g., textiles, contact lenses, or medical implants, intensive efforts to prepare a new class of materials with antibacterial activity are needed. Over the past decade, a variety of modifications or treatment techniques has emerged to obtain efficient antibacterial surfaces [1]. Polymeric materials with antibacterial properties are extensively studied in the form of a bactericidal polymer on its own [2,3,4,5,6], physical loading of a biocidal active substance (biocide) into a polymer [7,8,9,10,11,12,13,14,15,16], or a chemical functionalization with a bactericidal or bacteriostatic compound [17,18,19,20,21,22,23]. The antibacterial activity of biocide loaded in the polymer is caused by its diffusion, while surfaces modified with polycations kill bacteria through direct contact. Various cationic polymers show high levels of sustained antibacterial activity, as described in several reviews [24,25,26,27,28,29,30]. Examples of such polymers include poly(4-vinylpyridine) [4,31], polyethylenimine [32,33], and poly(*N*,*N*′-dimethylaminoethyl methacrylate) [3,34,35]. Functional amino groups of these polymers are often quaternized to enhance their bactericidal activity [31,34,36,37], as they are capable of interacting with the negatively charged cell walls of bacteria. The subsequent reaction with the cell membrane leads to its disorganization and the leakage of the intracellular material, followed by wall lysis due to autolytic enzymes [30].

Among these macromolecules, poly(*N*,*N*′-dimethylaminoethyl methacrylate) (PDMAEMA), which can be easily obtained using atom transfer radical polymerization (ATRP) [38] and quaternization, appears to be one of the most promising polymers for this type of application.

The linear topology of PDMAEMA was introduced to a solid support via “grafting to” [39,40,41] and “grafting from” [42,43,44,45] methods. The antimicrobial properties of linear polymers grafted to various materials, including filter paper [42], glass slides [42,46], silicon wafers [46], polypropylene [47], and others [44,48], were also studied. The obtained layers exhibited biocidal activity against *Escherichia coli* [42,44,46,47,48], *Bacillus subtilis* [42], and *Staphylococcus aureus* [44,48] strains. The accessibility of cationic groups in linear polymer materials may be limited, and their insufficient number may affect their antimicrobial activity, as the antibacterial properties strongly depend on the number of active cationic species [3].

The solution to this problem may be the use of star polymers, which due to their unique conformation and large number of functional groups, are well suited for this purpose. Therefore, we believe that nanolayers formed with the use of PDMAEMA stars will be an encouraging alternative to linear polycations. The use of star polymers as antimicrobial materials is quite limited. A covalent attachment of PDMAEMA stars to solid substrates is a novel method ensuring the stability of the layers. In the literature, there are only a few reports on thin films [3] and microfibers [34] composed of star polymers and noncovalent coatings of star polymers placed on a glass support [4,49,50].

Herein, we describe the covalent immobilization of the PDMAEMA stars to solid supports via a “grafting to” method by using UV irradiation to obtain stable nanolayers with biocidal activity and enabling their reuse. For comparison, layers made of linear PDMAEMA using the same “grafting to” method were also obtained in this research. The antibacterial activity of all synthesized layers was studied for either quaternized or nonquaternized layers made of PDMAEMA using a strain of Gram-positive bacteria *Bacillus subtilis* ATCC 6633. Research oriented toward a better understanding of the relationship between the topology and modification of the received layers and their antimicrobial properties enabled effective materials to be obtained that would be applicable in areas where the purity of health and human life is important.

## 2. Materials and Methods

### 2.1. Materials

1,2-Dichlorobenzene (99%), 1,1,4,7,10,10-hexamethyltriethylenetetramine (HMTETA, 97%), 2-bromopropionitrile (97%), 4-hydroxybenzophenone (98%), bromoethane (98%), allyl bromide (97%), copper(I) bromide (CuBr, 99.999%), copper(II) bromide (CuBr_2_, 99%), fluorescein sodium salt (>98%), hexadecyltrimethylammonium bromide (≥99%), p-xylene (≥99%), potassium carbonate (99.99%), and Pt-C catalyst were purchased from Sigma Aldrich (Poznan, Poland) and used as received. *N*,*N*′-dimethylaminoethyl methacrylate (DMAEMA, 98%), chlorodimethylsilane (DMCS, 98%), and triethylamine (>99%) were purchased from Sigma Aldrich (Poznan, Poland) and purified by distillation prior to use. A Dowex Marathon MSC ion exchanger was purchased from Sigma Aldrich (Poznan, Poland) and transformed into H^+^ using 1.6 M HNO_3_. Acetone (99.5%), chloroform (98.5%), diethyl ether (99.5%), hydrogen peroxide (H_2_O_2_, 30%), methanol (99.8%), sodium hydroxide (NaOH, 97%), and sulfuric acid (H_2_SO_4_, 95%) were purchased from POCH(Gliwice, Poland) and used as received. Toluene (99.5%) and tetrahydrofuran (THF, pure p.a.) were purchased from POCH (Gliwice Poland) and purified by distillation prior to use. Phosphate buffered saline (PBS) was purchased from PAA Laboratories GmbH (Pasching, Austria). 4-Allyloxybenzophenone was obtained according to the procedure described in [51].

Silicon wafers (p-doped, 100-oriented, 10–20 Ω cm resistivity, thickness of 505–545 μm) were purchased from Cemat Silicon S.A. (Warsaw, Poland) and cut into 10 × 10 mm pieces for modification. Glass wafers made of pure white borosilicate (diameter of 13 mm, thickness of 0.13 mm) were purchased from VWR International (Dresden, Germany). A strain of *Bacillus subtilis* ATCC 6633 was obtained from a collection from The Institute for Ecology of Industrial Areas (Katowice, Poland) on a liquid nutrient solution Luria–Bertani (LB) from A & A Biotechnology (Gdynia, Poland).

### 2.2. Synthesis of the N,N′-Dimethylaminoethyl Methacrylate Star and Linear Polymer

The synthesis and characterization of *N*,*N*′-dimethylaminoethyl methacrylate star polymers on a hyperbranched poly(arylene oxindole) macroinitiator (PArOx) were published elsewhere [52,53]. The synthesis of the linear PDMAEMA was performed by a standard ATRP procedure [54]. Briefly, for star synthesis, PArOx was used as the macroinitiator, while for the linear topology, 2-bromopropionitrile (monomer:initiator in a molar ratio of 1:1) was applied. The catalyst system for both syntheses was CuBr and HMTETA as a ligand; for stars, CuBr_2_ was also added. 1,2-Dichlorobenzene was used as a solvent. After the desired molar mass of the polymer was obtained, the solution in THF was passed through a column with a Dowex-MSC-1 ion exchange resin to remove the copper. Next, the resulting solution was dialyzed against methanol and then against water (SpectraPor membrane with MWCO 1000 g/mol) and dried by lyophilization.

### 2.3. Preparation of Benzophenone Modified Wafers

Silicon and glass wafers were modified to introduce photoreactive groups of 4-(3′-chlorodimethylsilyl)propyloxybenzophenone onto the surface. First, the wafers were properly cleaned as previously described [55,56]. Briefly, an acetone washed wafer was immersed in “piranha” solution (H_2_O_2_:H_2_SO_4_ 3:1 (*v*/*v*)) and heated at 100 °C for 2 h. After that time, the wafers were rinsed a few times with deionized water and acetone and dried under a vacuum.

4-(3′-Chlorodimethylsilyl)propyloxybenzophenone was synthesized according to a hydrosilylation procedure [51]. Briefly, 4-allyloxybenzophenone (2 g, 8.4 × 10^−3^ mol) and a Pt-C catalyst (10 g, 10% Pt) were suspended in 20 mL (17 g, 1.8 × 10^−1^ mol) of freshly distilled chlorodimethylsilane. The mixture was refluxed for 8 h in an oil bath at 40 °C. The obtained oily product was dissolved in toluene, and then, the catalyst was removed by filtration.

The cleaned wafers were immersed in a solution of 4-(3′-chlorodimethylsilyl)propyloxybenzophenone in toluene, and 3 mL (2.2 g, 2.2 × 10^−2^ mol) of triethylamine were added. The mixture with the wafers was left to stand overnight in the dark. After that time, the wafers were removed from the reaction mixture, rinsed a few times with chloroform, and dried in air [51].

### 2.4. Formation of Polymer Layers on the Glass and Silicon Wafers with UV Irradiation

The formation of layers made of DMAEMA polymer was performed by spin-coating linear (10% *v*/*v*) or star polymer (1% *v*/*v*) solutions in a mixture of acetone/THF (3:1 *v*/*v*) for 1 min on the silicon or glass wafers. A spin speed of 1500 rpm for the linear polymer and 2000 rpm for the star was applied. A mixture of THF and acetone at a volume of 1:3 (*v*/*v*) was used as the solvent. After the spin-coating process, the samples were dried in air and irradiated with UV light. For this purpose, a UVIlite LF 215S 2 × 15 W lamp was used at a wavelength of 254 nm (UVIlec, Cambridge, U.K.). The wafers were irradiated at a distance of 8 cm from the light source under argon atmosphere to avoid oxygen quenching. The irradiation time was 30 min, and after that, the samples were extensively rinsed with acetone to remove any residual impurities and unbound polymer. Then, the samples were dried in a vacuum and stored under an argon atmosphere [51]. The obtained layers were designated as SL1–SL4 for layers made of linear polymer and SG1–SG4 for layers made of star polymer with different molar masses.

### 2.5. Quaternization of the Linear and Star PDMAEMA

The conditions of the quaternization reaction were first determined for the polymers in solution and then transferred to the layers. For quaternization of PDMAEMA in the solution, 15 × 10^−3^ g of linear and star PDMAEMA (2 × 10^−2^ mol of amino groups, samples L2 and G2, Table 1) were dissolved in 10 mL of acetone. Next, 3.3 g (3 × 10^−2^ mol, 2.2 mL) of ethyl bromide were added (ethyl bromide:amino groups in a molar ratio of 1.5:1). The reaction was performed in a flask under atmospheric conditions. The flask was placed in an oil bath at 40 °C, and the mixture was kept on a magnetic stirrer for 24 h. After this time, the mixture became cloudy and was left to settle. Then, the acetone was decanted, and the polymer was washed several times with fresh acetone (20 mL each). Next, the quaternized polymers in acetone were dialyzed against pure water for 2 days (Roth membrane, regenerated cellulose, MWCO 4000–6000 Da) and dried by lyophilization.

For quaternization of the PDMAEMA layers, silicon and glass wafers with immobilized polymers (samples SL2, SL4, SG2, and SG4) were placed in 250 mL of acetone, and then, 73.5 g (67 × 10^−2^ mol, 50 mL) of ethyl bromide were added (ethyl bromide:amino groups of PDMAEMA in a molar ratio of 1.5:1), as the procedure given in [46,57]. The wafers were left in solution with a magnetic stirrer in an oil bath at 40 °C for 24 h. Next, the samples were extensively rinsed with distilled water to remove residual impurities, dried in a vacuum, and stored under an argon atmosphere. The obtained layers were designated QSL2 and QSL4 for layers made of linear polymer and QSG2 and QSG4 for layers made of stars.

The quantity of quaternary groups in the polymer structure was measured by UV-Vis spectroscopy by measuring the amount of bonded fluorescein to the PDMAEMA amino groups, according to the procedure described in the literature [46,57]. In the measurements, it was assumed that one fluorescein molecule corresponded to one quaternary group of the attached polymer.

For this purpose, the wafers with PDMAEMA star polymers (samples SG2 and SG4) and linear polymers (SL2 and SL4) were immersed in 1 wt% fluorescein sodium salt aqueous solution for 10 min. Afterwards, they were rinsed several times with water, placed in 3 mL of 0.1 wt% aqueous solution of hexadecyltrimethylammonium bromide, and shaken for 20 min at 300 rpm on an orbital shaker to rinse the dye bonded with the quaternized groups. A 10% *v*/*v* solution of 100 mM phosphate buffered saline (PBS, pH 7.4) was added to the shaken solution, and then, the absorbance of the resultant aqueous solution was directly measured at λ = 501 nm.

### 2.6. Evaluation of Antibacterial Properties

A determination of the bactericidal activity was performed against *Bacillus subtilis* ATCC 6633 using the linear polymer layers (samples SL4, QSL4) and star polymer layers (samples SG4 and QSG4) immobilized on silicon wafers.

The strain of *Bacillus subtilis* ATCC 6633 was preserved at −70 °C in a Luria–Bertani (LB) medium supplemented with 20% *v*/*v* glycerol. Bacterial cultures that were 24 h old were obtained from the LB medium and adjusted to OD_600nm_ = 0.8 (approximately 10^7^–10^8^ CFU/mL) and were introduced as an inoculum to 100 mL Erlenmeyer flasks containing 20 mL of the LB medium. The cultures were grown aerobically for 24 h with constant shaking (120 rpm) at 30 °C. After incubation, the bacterial culture was centrifuged at 10,000× *g* for 10 min. The obtained biomass was washed two times with deionized water and finally suspended in 10 mL of sterile saline solution to a concentration of 10^6^ CFU/mL.

Then, PDMAEMA layers were immersed in cell suspensions. The silicon wafer without the polymer was used as a control.

The samples were incubated at 30 °C and analyzed after 5 min, 60 min, and 24 h.

The number of bacterial cells was determined by a serial dilution method. All experiments were performed in triplicate and analyzed individually.

For this purpose, the cell suspension from a conical tube was diluted and placed on an LB plate. The plates were incubated at 30 °C for 24 h.

After the incubation period, the number of colonies grown on the plate was counted, and the result was given as colony forming unit (CFU)/mL (1). The results from two replicates were averaged.

(1) $$\frac{CFU}{mL}\;in\;the\;tested\;material=\frac{number\;of\;colonies}{\left(dilution\;factor\right)\cdot \left(volume\;of\;the\;culture\;plate\right)}$$

### 2.7. Characterization Methods

Gel permeation chromatography with multiangle laser light scattering detection (GPC-MALLS) was used to determine the molar mass and molar mass distributions of the linear and star PDMAEMA. Analysis was performed in DMF containing 5 mmol/L lithium bromide at 45 °C with a nominal flow rate of 1 mL/min. A column set containing GRAM columns from Polymer Standards Service (PSS): guard + 100 Å + 1000 Å + 3000 Å was used. A differential refractive index detector (Δn-2010 RI WGE Dr. Bures) and a multiangle laser light scattering detector (DAWN HELEOS from Wyatt Technologies) were used in the system. The results were evaluated with ASTRA 5 software (Wyatt Technologies, Santa Barbara, CA, USA).

NMR measurements were performed using a Bruker Ultrashield 600 spectrometer (600 MHz for ^1^H). NMR spectra were recorded in ppm and referenced to the tetramethylsilane (TMS) peak.

Atomic force microscopy (AFM) was performed in tapping mode with a MultiMode AFM microscope equipped with a NanoScope 3D controller (Veeco Instruments Inc., New York, NY, USA) and 125 nm single crystal silicon cantilevers (Model TESP, Veeco Instruments Inc.) for polymer layers immobilized on glass layers. The measurements were carried out in air with different scan sizes (from 500 nm × 500 nm to 10 μm × 10 μm). NanoScope software was used to record images at different surface points. For each sample, the root mean squared roughness (RMS) was appointed and averaged from a few measurements of different cross-sections of the images. The average roughness (RMS) was calculated from a 2 × 2 µm area. An approximate layer thickness was estimated by scratching the layer made of polymer on the surfaces and measuring the depth of the crack formed.

The thickness of the layers made of DMAEMA polymer on the silicon wafers was examined by ellipsometry. The analyses were performed using an SE 850E spectrometer (Sentech, Krailling, Germany) in a wavelength range of 240–2500 nm controlled by SpectraRay 3 software. The measurements were performed on dry surfaces using the Cauchy method. The thickness of the polymer layers was calculated on the basis of a multilayer model that included SiO_2_ on a silicon wafer, a hydroxylated wafer, and a benzophenone derived layer.

The amounts of quaternary amines were determined by a Jasco V-530 UV-Vis spectrophotometer (Oklahoma, OK, USA) equipped with a programmable Medson MTC-P1 thermocontroller. The measurements were made based on a standard curve (previously designated), and a wavelength of λ = 501 nm was used.

The visualization of the bacteria on the star polymer layers that were immobilized on the silicon wafers was characterized using scanning electron microscopy (SEM, FEI Company, Hillsboro, Oregon, USA, Quanta 250 FEG). The micrographs were obtained under low vacuum (80 Pa) with an acceleration voltage of 10 kV from secondary electrons collected by a large field detector (LFD). Solid samples were stuck to microscopic stubs by double sided adhesive carbon tape.

X-ray photoelectron spectroscopy (XPS) was used to analyze the quantitative chemical composition of the polymer structure on the silicon surfaces. The measurements were performed using a PHI5700/660 (Physical Electronics, Chanhassen, MN, USA) spectrometer with 1486.6 eV monochromatic Al-Kα radiation (energy resolution of 0.3 eV). To obtain information regarding the chemical composition and atomic concentration of the samples, the survey spectra and the core lines for the C 1s, O 1s, Si 2p, N 1s, and Br 3d electronic states were measured. The atomic concentrations were calculated using Multipak software (ver. 9.7.0). The fitting process was applied to the core levels using SimPeak software (ver. 2.1).

The mass spectra and distribution maps of specific ions detected from examined surfaces were obtained using a secondary ion spectrometer ToF-SIMS V (ION-TOF GmbH, Münster, Germany). For realized measurements, the secondary ions were measured in the mass range 1–1000 Da using a primary ion beam (Bi_3_^+^, *I* = 0.20 pA) focused on area of 300 × 300 μm. In order to remain in the static regime of the technique, the spectra were collected keeping low resolution of the data points using a 128 × 128 pixel raster size and maintaining the dose of bismuth ions per cm^2^ at the level below 10^12^ ions per cm^2^. The calibration of mass spectra was performed with respect to H^+^, H_2_^+^, CH_2_^+^, C_2_H_3_^+^, C_3_H_3_^+^, and C_3_H_5_^+^ peaks. The distribution maps were reconstructed from the mass spectra. The measurements and further analysis were performed using SurfaceLab 6.4 software (Kirkland, WA, USA).

## 3. Results and Discussion

### 3.1. Formation of the Layers Made of the Star and Linear Polymers on a Solid Surface

**Scheme 1:**
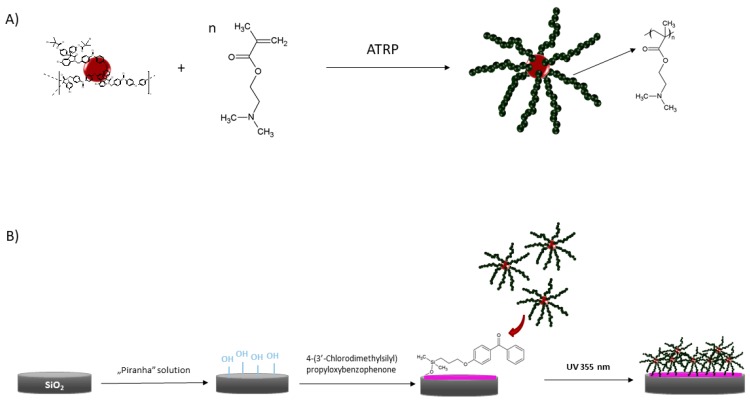
Preparation of the star polymer layers. (**A**) Synthesis of star polymers via the “core-first” method. (**B**) Immobilization of the polymer on the solid support. ATRP, atom transfer radical polymerization.

The “grafting to” method, consisting of the binding of polymer chains to solid surfaces, enables the preparation of layers with precisely planned polymer structures and provides stable attachment of the polymer to the substrate via covalent coupling between suitable functional groups [39,40,41]. Herein, the linear and star polymers of *N*,*N*′-dimethylaminoethyl methacrylate were immobilized on a benzophenone functionalized solid surface via a “grafting to” approach. In contrast to the common chemical procedures, where the functionalization of polymer is necessary for its covalent binding to the substrate, this method does not require tedious synthetic efforts. Additionally, the photochemistry of the benzophenone moieties is well known and can be applied in many chemical environments. The photoreactive groups bonded to solid support ensure the chemical inertness of the substrate in the absence of light. Due to the above mentioned reasons, it is a simple and effective method to attach a polymer to a solid substrate covalently. The route to layers made of DMAEMA star polymers is presented in Scheme 1.

First, the star polymers were obtained via atom transfer radical polymerization (ATRP) using the “core-first” method, which enables the use of core initiating groups to obtain structures of a desired arm number and length (Scheme 1A). Four stars were selected for immobilization. They consisted of a poly(arylene oxindole) core and twenty-eight PDMAEMA arms with degrees of polymerization equal to 86, 63, 109, and 222 (samples G1–G4, Table 1). The linear poly(*N*,*N*′-dimethylaminoethyl methacrylate) analogues were obtained via ATRP for comparison of the layer formation process by different topologies (samples L1–L4, Table 1). The molar masses and molar mass distribution (M_w_/M_n_) of the polymers were determined using gel permeation chromatography with multiangle laser light scattering detection (GPC-MALLS) and are given in Table 1. The molar masses of the linear polymers were chosen to correspond to the molar mass of a single star arm (L1 corresponds to G1, L2 to G2, L3 to G3, and L4 to G4, respectively).

Two types of solid substrates were used for polymer immobilization: glass and silicon. The glass and silicon wafers could be easily modified and were resistant to the chemicals used during the modification. The choice of substrate was dependent on the layer characterization method. The functionalization and polymer attachment on both types of substrates was carried out as shown in Scheme 1B. First, the glass and silicon wafers were properly modified in “piranha” solution to obtain reactive hydroxyl groups on the surface. Hydroxyl groups allowed the introduction of 4-(3’chlorodimethylsilyl)propyloxybenzophenone to the surface. In the next step, the polymers were deposited on silyl modified surfaces by spin-coating from the THF/acetone (1:1 *v*/*v*) solution. Then, the polymer coated supports were irradiated with UV light. Irradiation led to the formation of radicals in the benzophenone ketone groups, followed by their reaction with the carbon atoms in the polymer chain. This process enabled the covalent attachment of the PDMAEMA to the surface. After the irradiation process, the layers were thoroughly rinsed with acetone, which guaranteed that only the polymers bound to the supports were analyzed in further experiments.

### 3.2. Physicochemical Characterization of the Obtained PDMAEMA Layers

Changes in the morphology and chemical composition of the surfaces were monitored using AFM, XPS, and ToF-SIMS techniques after every step of wafer functionalization and polymer immobilization.

**Figure 1:**
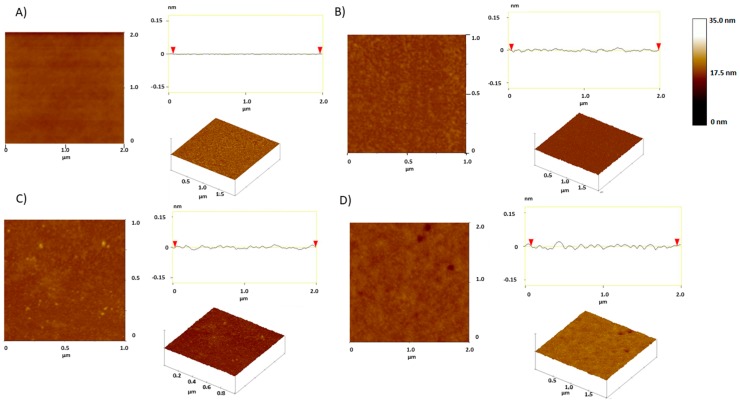
Atomic force microscopy images: (**A**) bare wafer with -OH group, (**B**) wafer with benzophenone derivative, (**C**) wafer with immobilized linear PDMAEMA (sample L3, Table 1), and (**D**) wafer with immobilized star PDMAEMA (sample G3, Table 1).

AFM images of the obtained surfaces are shown in Figure 1. The value of the root mean squared (RMS) deviation of surface roughness after each step of preparation increased. The increase from approximately 0.1 nm after the hydroxylation process, through 0.4 nm for the benzophenone modified surface, to 0.55 and 0.67 nm for the linear and star polymers, respectively, was observed. The RMS values showed that the layer made of the star polymer was rougher than the layer made of the linear structure, as was visible in the AFM images (Figure 1C,D).

Quantitative XPS measurements were used to determine the elemental composition of the wafers after various stages of modification. The linear and star polymer layers immobilized on silicon substrates were also characterized.

The changes in the atom concentrations on the silicon substrate after hydroxylation (sample SOH), after benzophenone functionalization (sample SBPH), and linear or star polymer immobilization (SL3 and SG3, Table 1, respectively) are shown in Table 2.

The carbon to oxygen (C/O) ratio increased from 0.2% for the SOH surface to 0.4% for the SBPH, which confirmed the functionalization of the wafer with a benzophenone derivative. The evidence of the covalent attachment of the polymer to the surface was the strong increase in the C/O ratio after the immobilization process. The increase was more visible for the star topology layer than for the linear layer, which was probably caused by the comparably much higher molar mass of the star used for immobilization. After the final creation of the star structure layer, a small atomic concentration of approximately 0.3% of bromine coming from the star polymer was detected. Additionally, the atomic concentration of nitrogen increased for both types of topologies in comparison with the SOH and SBPH wafers due to the appearance of the PDMAEMA amino groups.

**Figure 2:**
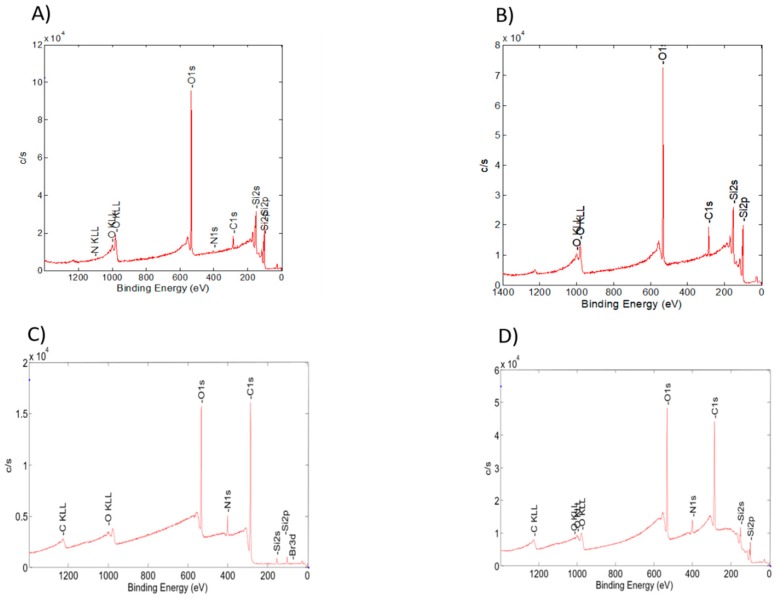
XPS survey spectra of (**A**) the OH terminated surface, (**B**) the BPH terminated surface, (**C**) the star polymer layer (SG3, Table 2), and (**D**) the linear polymer layer (SL3, Table 2).

The survey photoemission spectrum of the SOH wafers contained oxygen, carbon, and silicon lines (Figure 2A), with the highest intensity for the oxygen signal (O 1s). The carbon signal observed on the XPS spectrum probably originated from hydrocarbon and carbon dioxide surface contaminants. These compounds are considered a standard impurity in XPS analysis and are routinely used as internal standards to calibrate peak positions [58]. Since this contamination had no effect on further surface modifications or on the antimicrobial properties, no attempt was made to remove it. After the benzophenone-silylation process (sample SBPH, Figure 2B), the intensity for the carbon signal (C 1s) increased, while oxygen decreased. The formation of the layers made of both polymer architectures (samples SL3 and SG3, Table 2) caused an increase in the carbon intensity of (C 1s), and characteristic shifts for silicon, carbon, oxygen, and nitrogen were visible (Figure 2C,D). In the case of the both polymer layers, additional signals appeared corresponding to the bromine atoms (Br 3d) (Figure 2C,D). These signals were better visible at deconvoluted lines of XPS spectra (Supporting Information, Figure S1A,B). Bromine atoms originated from the PArOx core and from the ends of the arms in case of star layers and only from the ends of PDMAEMA chains for linear layers [59]. For comparison, the benzophenone functionalized layers (sample SBPH) did not show the presence of bromine at all (Supporting Information, Figure S1C).

**Figure 3:**
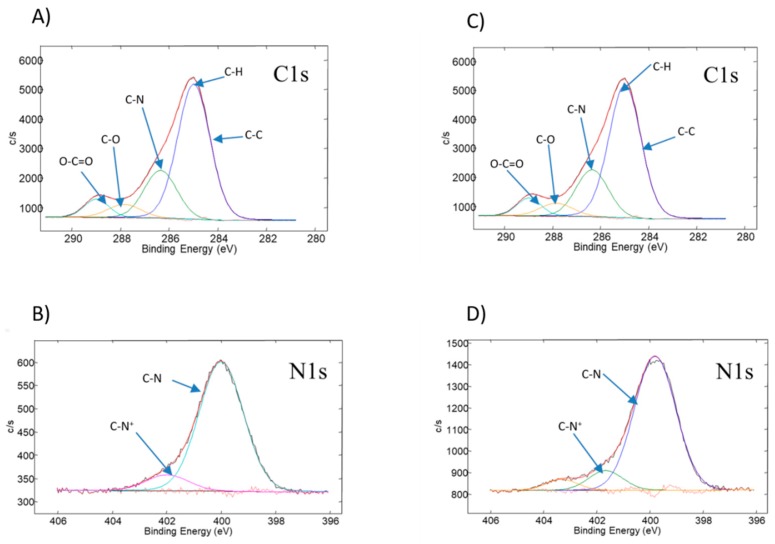
The deconvoluted lines of XPS spectra: (**A**) the C 1s core level of the star polymer layer, (**B**) the N 1s core level of the star polymer layer (sample SG3, Table 2), (**C**) the C 1s core level of the linear polymer layer, and (**D**) the N 1s core level of the linear polymer layer (sample SL3, Table 2).

Figure 3 presents the deconvoluted lines of the C 1s and N 1s core levels for the star and linear polymer layers. The C 1s and N 1s core levels deconvolution lines spectra after hydroxylation (sample SOH) and after benzophenone derivative functionalization (sample SBPH) are shown in Figure S2 (Supporting Information). The observed lines confirmed successful surface functionalization.

In the high resolution C 1s spectra of the star layer, four characteristic peaks were observed, corresponding to C-C, C-H, C-N, C-O, and O-C=O bonds, which were detected at binding energies of 285, 284.7, 286.4, 287.7, and 289 eV, respectively (Figure 3A). Additionally, the N 1s spectra recorded for this layer exhibited two peaks of neutral C-N at 400 eV and C-N^+^ at 402 eV, which might be assigned to amine groups of the cationic polymer (Figure 3B). Similar binding energy values were observed for layers made of linear PDMAEMA (Figure 3C,D).

The appearance of all these species confirmed the successful covalent modification of silicon wafers with both PDMAEMA topologies.

**Figure 4:**
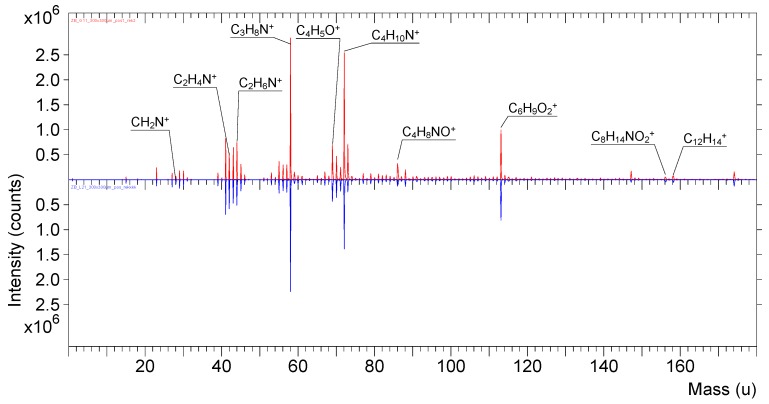
Mass spectra of the SG3 (red) and SL3 (blue) samples collected in positive polarity. The intensity scale of mass spectra of the SL3 sample is reversed for easier comparison of the intensities of a particular ion.

Time of flight secondary ion mass spectrometry (ToF-SIMS) is an analytical method that delivers information about the molecular and elemental species present on a surface. The positive ToF-SIMS spectra of star and linear PDMAEMA layers are shown in Figure 4.

The characteristic signals derived from secondary ions such as CH_2_N^+^ (m/z = 28), C_2_H_4_N^+^ (*m*/*z* = 42), C_2_H_6_N^+^ (*m*/*z* = 44), C_3_H_8_N^+^ (*m*/*z* = 58), C_4_H_5_O^+^ (*m*/*z* = 69), C_4_H_10_N^+^ (*m*/*z* = 72), C_4_H_8_NO^+^ (*m*/*z* = 86) C_6_H_9_O_2_^+^ (*m*/*z* = 113), C_8_H_14_NO_2_^+^ (*m*/*z* = 156), and C_12_H_14_^+^ (*m*/*z* = 158) were identified as fragments of the PDMAEMA chains. The presence of this signals confirmed the successful covalent immobilization of the PDMAEMA stars, as well as the linear PDMAEMA to the solid substrate.

**Figure 5:**
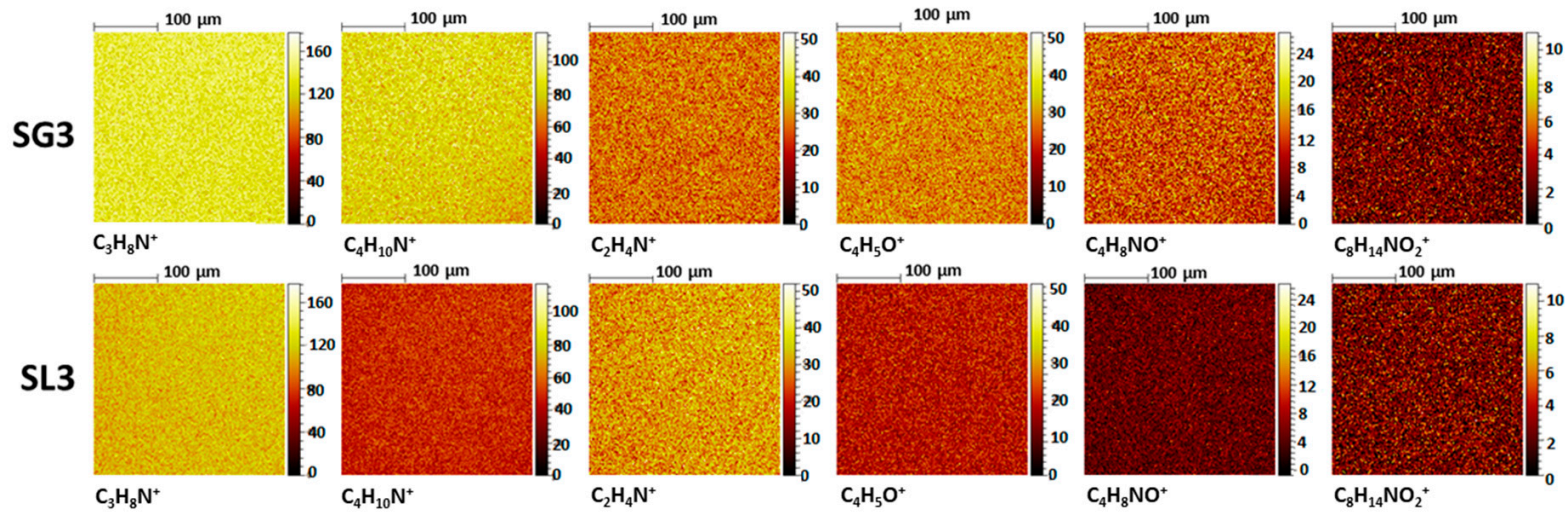
Distribution maps of selected ions obtained from the 300 × 300 μm areas for the SG3 (first line) and SL3 nanolayers (second line). The intensity scale of distribution maps was unified within a particular ion.

The ToF-SIMS distribution maps of ions characteristic for PDMAEMA chains are shown in Figure 5. The distribution of particular ions was highly homogeneous for both types of nanolayers.

The thickness of the resulting layers made of DMAEMA polymers was established using ellipsometry and AFM measurements (Table 3). In the latter method, the measured thickness value was the depth of the scratched “hole” by the needle in the layer made of polymer. The average value taken from the depth of three scratched holes is collected in Table 3.

The UV sensitive compound formed a nanolayer with a thickness of approximately 1–2 nm as measured by ellipsometry. A similar value was obtained by Prucker et al. [51]. After the UV mediated immobilization of the polymers, the dependence of their molar mass, structure, and concentration on layer thickness was studied.

First, the effect of the polymer concentration in the mixture of acetone and THF, used during the spin-coating process, on the layer thickness was checked on the exemplary L2 and G2 polymer nanolayers on the silicon wafers (Supporting Information, Table S1).

For linear polymers, the highest concentration above which no changes in the thickness of the linear nanolayer was observed was 10 wt%, while for the star polymer, it was equal to 1 wt%.

At lower concentrations than mentioned above, the polymers did not form a uniform layer because the applied solution was too diluted. When the concentration exceeded these values, the thickness of the obtained linear polymer nanolayers remained unchanged. Above a 60 wt% concentration of the SL2 polymer and a 1 wt% concentration of the SG2 polymer, the solution was too viscous to form the layer.

Therefore, a concentration of 10 wt% for the linear polymer and 1 wt% for the star was used for further studies. The results obtained from both AFM and ellipsometry were similar for nanolayers made of linear polymers. For layers formed from star polymers, slight discrepancies between both techniques were visible (Table 3). It seemed that as the layer thickness increased, the influence of the measuring technique became more pronounced. Ellipsometry used the Cauchy mathematical model for thickness calculation, while the AFM method thickness was determined as the average depth of scratched “holes”.

As expected, for both linear and star topologies, an increase in the thickness was observed with an increase in the molar mass of immobilized polymers. Based on the obtained values and on the hydrodynamic diameters of the star polymers in acetone, which were in the range of 20–30 nm [60], it could be concluded that stars were formed not as monolayers, but rather in the form of multilayers. A similar situation was observed for layers made of stars with poly[oligo(ethylene glycol) methacrylate] arms where the obtained layer thickness made of the star polymer was in the range of 58–59.5 nm [59].

The largest thickness of 120 nm was obtained for the layer of the star polymer with the highest molar mass (sample SG4, Table 3). The obtained values indicated that star polymers formed much thicker layers than those formed from their linear analogues with molar masses comparable to the arms of the stars. The probable reason for such results was that the layers were composed of interpenetrating star polymer arms, and through the large packing of star macromolecules and their close distance to each other, they formed thicker multilayers. Furthermore, during the UV irradiation of the polymers, the recombination of the radicals between the arms of the stars occurred, causing cross-linking. Additionally, the stars contained a poly(arylene oxindole) core, which possessed a similar UV reactive group as the benzophenone derivative in the chemical structure. If the radicals were also created in the core of the stars, crosslinking between the star macromolecules was also possible, causing an additional increase in the layer thickness.

### 3.3. Quaternization of the PDMAEMA Nanolayers

The pendant amino groups in the obtained nanolayers were modified with ethyl bromide for their quaternization. This process is known to increase the antibacterial properties of PDMAEMA through enabling stronger interactions with the bacterial wall, which facilitates bacterial destruction.

First, the reaction conditions were optimized for polymers directly dissolved in the solution. For this purpose, both PDMAEMA topologies (samples L2 and G2, Table 1) were quaternized in acetone with ethyl bromide, yielding QPDMAEMA bromide salts (samples QL2 and QG2). The 100% yield of the quaternization process was verified by ^1^H NMR spectroscopy (Supporting Information, Figure S3).

**Scheme 2:**
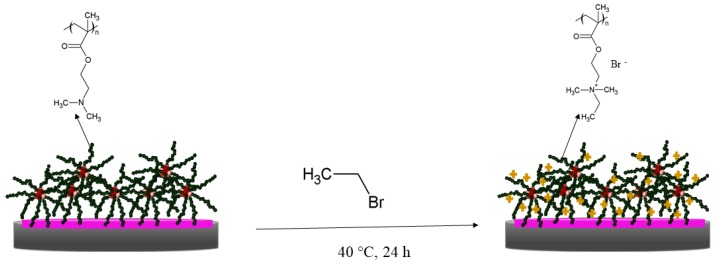
The preparation of quaternized PDMAEMA nanolayers.

The obtained PDMAEMA nanolayers (samples SL2, SL4 and SG2, SG4, Table 3) were quaternized by their immersion in acetone containing ethyl bromide in a 1:5 volume ratio and subsequent heating to 40 °C (Scheme 2). The concentration of the quaternized groups at the surface was measured by a colorimetric method, based on their complexation with fluorescein sodium salt, as described for quaternized PDMAEMA brushes grafted on magnetic nanoparticles [61]. For this purpose, first, the standard curve of fluorescein dye solution in water was performed (Supporting Information, Figure S4). Next, the polymer containing wafers were immersed in the dye solution to bind the fluorescein to the amino groups. After washing several times with water, the plates were immersed in a hexadecyltrimethylammonium bromide surfactant, which caused the dye to desorb back into the solution. Then, the solution was tested by UV-Vis spectroscopy to determine the concentration of the washed dye. According to the reaction mechanism, one mole of fluorescein sodium salt was assumed to correspond to one mole of quaternized amino groups per cm^2^.

The quantification results of the quaternized amino groups for the star polymer nanolayers QSG2 and QSG4 and for QSL2 and QSL4 immobilized on the silicon wafers are collected in Table 4. The use of star polymers in comparison with linear polymers in layer formation significantly increased the concentration and quantity of the quaternized amino groups per cm^2^ of the surface. As expected, the molar mass of the polymer used also increased the quantity of quaternary groups per cm^2^ of the surface, independent of the topology of the macromolecules.

**Figure 6:**
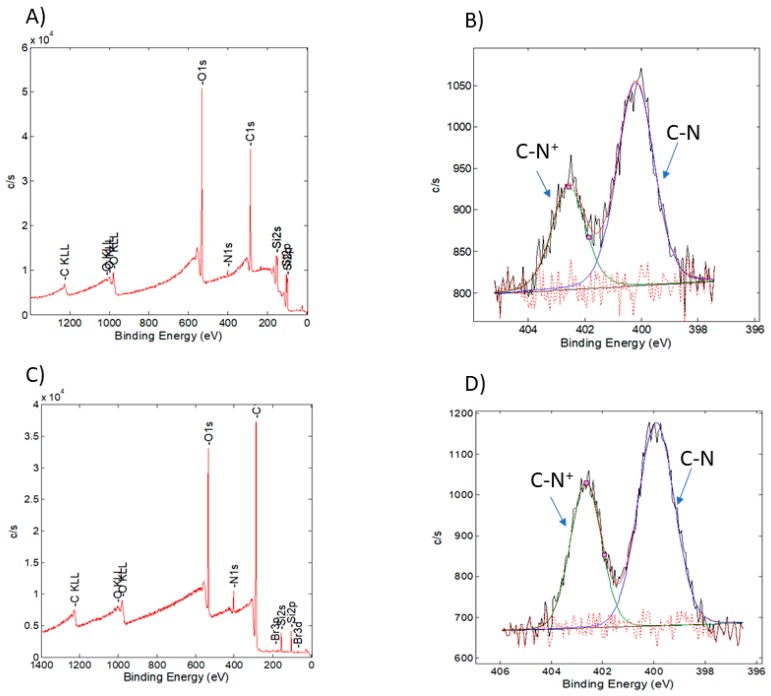
XPS spectra of the QPDMAEMA nanolayers: (**A**) survey spectra and (**B**) the N 1s core level of the linear QPDMAEMA nanolayer (sample QSL4, Table 4); (**C**) survey spectra and (**D**) the N 1s core level of the star QPDMAEMA nanolayer (samples QSG4, Table 4).

The quaternization of the PDMAEMA nanolayers was also confirmed using XPS spectroscopy. Figure 6 shows the XPS survey spectra of quaternized PDMAEMA nanolayers (samples QSL4 and QSG4, Table 4). After N-alkylation (QPDMAEMA structure), the C N^+^ peak component intensities for both topologies significantly increased compared to the PDMAEMA structures shown in Figure 3, which indicated that the amino groups of DMAEMA units were successfully quaternized. A similar increase in C N^+^ peak intensity was observed for the quaternized linear PDMAEMA grafted onto graphene oxide [62].

The obtained UV-Vis and XPS spectroscopy results confirmed that stable nanolayers made of the linear and star DMAEMA polymer were successfully prepared and that the process of N-alkylation was successfully completed.

### 3.4. Antibacterial Activity of PDMAEMA and QPDMAEMA Nanolayers

An assessment of the sensitivity of microorganisms to antibacterial substances is one of the essential stages of diagnostics. Sensitivity tests indicate the ability of an antibacterial agent to inhibit the growth of microorganisms in vitro. Currently, many methods are used to determine the level of antibacterial properties. One of the methods used is the so-called “dynamic contact condition” method, in which a substance with a potential antibacterial effect is directly in contact with a liquid bacterial culture. For this purpose, bacteria are propagated in a liquid medium, which are then incubated with a suitable agent. Growth inhibition is determined through the estimation of bacterial growth by a serial dilution method, according to the procedure described for quaternized PDMAEMA brushes grafted on magnetic nanoparticles [61].

A reference strain *Bacillus subtilis* ATCC 6633 was chosen as a model organism in the experiments. This strain is frequently used in laboratory studies for the evaluation of antibacterial activity against Gram-positive spore forming bacteria. The antibacterial properties of the star and linear PDMAEMA, as well as the quaternized and nonquaternized forms (samples SG4, QSG4, SL4, and QSL4, Table 3 and Table 4) immobilized on silicon wafers were examined. The layers were chosen for bacterial growth inhibition studies due to having the highest molar masses of the immobilized star and linear polymers; hence, the amount of functional (cationic) groups in the case of the quaternized polymers was the highest. As a control, bare silicon wafers were used. The results from a “dynamic contact condition” method are shown in Table 5.

Bacterial growth inhibition studies were conducted directly after the exposure of surfaces with PDMAEMA to *Bacillus subtilis.* The results showed that a layer composed of quaternized stars (sample QSG4, Table 5) exhibited a higher affinity for the inhibition of bacterial growth after direct contact compared with that of a nonquaternized layer (sample SG4, Table 5). Additionally, the layer composed of quaternized stars after 5 min of exposure to bacteria exhibited a higher biocidal effect compared with that of the layer of quaternized linear PDMAEMA. The most effective bactericidal abilities of the layer made of QSG4 (93% inhibition of bacterial growth) may be a result of having the highest amount of quaternary groups among all obtained layers (see Table 4) and their good accessibility to interacting with bacteria due to the star topology of the immobilized polymer.

**Figure 7:**
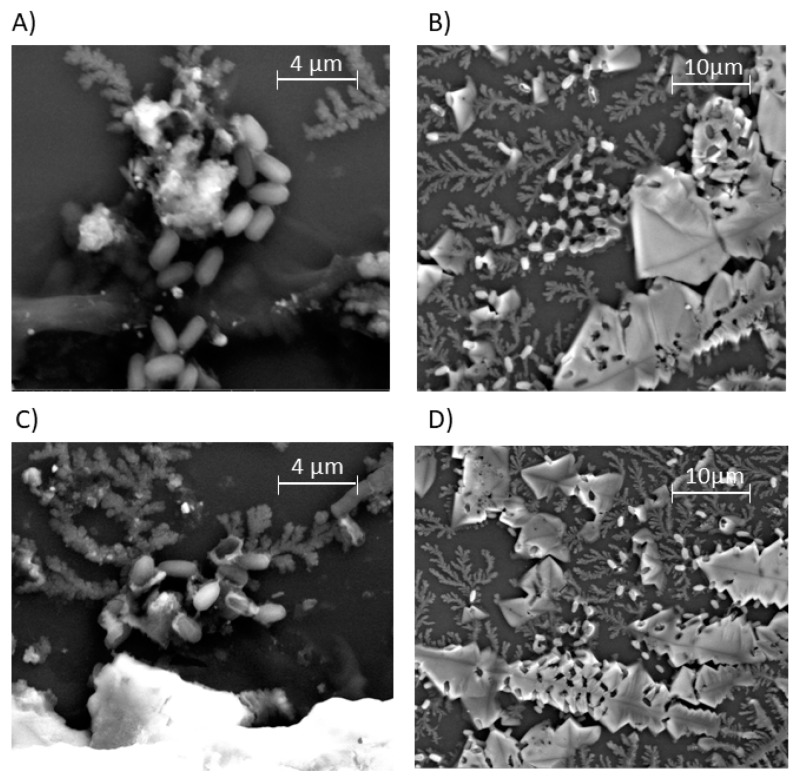
SEM microscopy images of quaternized PDMAEMA nanolayers after 24 h of exposure to bacteria with visible bacterial cells: (**A**,**B**) star nanolayers (sample QSG4, Table 5); (**C**,**D**) linear nanolayers (sample QSL4, Table 5).

After 1 h of the QSG4 star nanolayer exposure to bacteria, a decrease to 49% growth inhibition was observed, while QSL4 linear nanolayers exhibited 0% growth inhibition (Table 5). Such behavior was probably caused by the structure of the obtained layers, which enabled the strong interaction of the interpenetrating charged chains with bacteria. Most likely, bacterial cells settled on the quaternized layers, strongly penetrated the layer, and remained in its recess, thus covering the active polymer surface. The presence of bacterial cells on QPDMAEMA layers after 24 h exposure was additionally confirmed by SEM analysis (Figure 7).

To recover the antibacterial properties of such layers, the bacterial cells must be removed. For this reason, different methods can be used, such as mechanical scraping of the biofilm or washing it off by the use of solvents or detergents [42,44,63].

On the other hand, in the case of layers made of SG4 stars, the inhibition of bacterial growth (78% inhibition of bacterial growth) directly after exposure to *Bacillus subtilis* was not as pronounced as in the case of the QSG4 layer (93% inhibition of bacterial growth). The biocidal effect of nonquaternized SG4 star layers in the long term exposure was increased compared with QSG4 and was much higher than for nonquaternized SL4 linear layers.

Another situation was observed for layers composed of linear PDMAEMA. Directly after exposure to bacteria, the layers composed of quaternized linear polymer (sample QSL4, Table 5) not only exhibited decreased antimicrobial activity compared with the quaternized stars (79% inhibition of bacterial growth), but also lost this activity completely, after 1 h of exposure. In the case of layers composed of nonquaternized linear polymer (sample SL4, Table 5), the biocidal effect was observed to decrease from 88 to 62%, in contrast to the layers of nonquaternized star polymer (sample SG4, Table 5), where this effect increased in a long term treatment.

The most promising biocidal properties against *Bacillus subtilis* in the “dynamic contact condition” method were obtained for layers made of PDMAEMA composed of quaternized stars. However, due to bacterial cells settling on these layers, the long term biocidal effect was not satisfactory. It is important to investigate methods for washing away settling bacterial cells on the surface. These investigations are in progress. Our intention is to use various bacterial strains to evaluate the antibacterial properties of the synthetized materials.

## 4. Conclusions

Stable nanolayers made of star and linear PDMAEMA were obtained by covalent bonding of the polymers to a photoreactive benzophenone group immobilized on glass or silicon wafers. The polymer structures were covalently attached to a solid surface by UV light, which enabled a high density of polymer chains and provided stable covalent linkage between the polymer and solid support. The stars formed much thicker layers (50–100 nm) than the linear polymers (3–10 nm). The thickness of the star nanolayers indicated the formation of a multilayer morphology caused by the reaction not only with the support, but also by the recombination of radicals between densely packed star arms and cores. The obtained layers were successfully quaternized with ethyl bromide. For layers made of star polymer, the quantity of the quaternized amino groups was much higher than for linear polymers per cm^2^ of the surface and was dependent on the molar mass of the polymer; furthermore, the quantity of quaternized amino groups increased with molar mass regardless of the polymer topology. Our results confirmed that the nanolayers with the star topology of PDMAEMA promoted higher inhibition growth of *Bacillus subtilis* ATCC 6633 when in direct contact than the layers of linear PDMAEMA. The best results were obtained for quaternized layers of the star polymers, but a strong interaction between the surface and bacteria inhibited the long term antibacterial activity of the layers. The obtained results demonstrated that the star topology together with the modification of PDMAEMA increased the antibacterial activity of the PDMAEMA nanolayers and sufficiently inhibited bacterial growth.

## Figures and Tables

**Table 1:** The molar masses of the polymers used in the studies (L, linear; G, star). PDMAEMA, poly(*N*,*N*′-dimethylaminoethyl methacrylate); GPC-MALLS, gel permeation chromatography with multiangle laser light scattering detection.

PDMAEMA	M_n_ GPC-MALLS (g/mol)	M_w_/M_n_
L1	9000	1.09
L2	13,000	1.10
L3	16,000	1.08
L4	40,000	1.12
G1	320,000	2.70
G2	400,000	2.90
G3	560,000	2.33
G4	1,000,000	1.79

**Table 2:** Surface composition after each step of modification determined using XPS. SOH, silicon substrate after hydroxylation; SBPH, silicon substrate after benzophenone functionalization.

Sample	C 1s (%)	N 1s (%)	O 1s (%)	Br 3d (%)	C/O
SOH	18.3	1.9	79.8	0.0	0.2
SBPH	28.3	0.2	71.5	0.0	0.4
SL3	65.9	5.0	29.0	0.1	2.3
SG3	74.9	5.1	19.7	0.3	3.8

**Table 3:** Thickness of the PDMAEMA nanolayers as measured by AFM and ellipsometry.

Layer	Layer Thickness-AFM (nm)	Layer thickness-Ellipsometry (nm)
SL1	4	3
SL2	6	4
SL3	7	6
SL4	13	10
SG1	31	50
SG2	78	70
SG3	70	87
SG4	120	100

**Table 4:** Concentration and quantity of quaternized amino groups on the polymer nanolayers.

Nanolayers	Concentration of Quaternized Amino Groups on the Surface (mg/mL/cm^2^)	Quantity of Quaternized Amino Groups on the cm^2^ Surface
QSL2	0.005	2.40 × 10^16^
QSL4	0.031	1.49 × 10^17^
QSG2	0.323	1.55 × 10^18^
QSG4	0.463	2.22 × 10^18^

**Table 5:** Growth inhibition of *Bacillus subtilis* after incubation with PDMAEMA nanolayers for exposure times equal to 5 min, 60 min, and 24 h.

Nanolayer	Number of Bacteria (CFU/mL) 5 min	Growth Inhibition (%) 5 min	Number of Bacteria (CFU/mL) 60 min	Growth Inhibition (%) 60 min	Number of Bacteria (CFU/mL) 24 h	Growth Inhibition (%) 24 h
SG4	1.1 × 10^6^	78%	1.01 × 10^6^	80%	6.1 × 10^5^	89%
QSG4	3.3 × 10^5^	93%	2.5 × 10^6^	49%	7.2 × 10^6^	0%
SL4	6.2 × 10^5^	88%	6.9 × 10^6^	81%	2.4 × 10^6^	62%
QSL4	1.1 × 10^6^	79%	1.6 × 10^7^	0%	1.4 × 10^6^	0%
Control	5 × 10^6^	-	5.2 × 10^6^	-	6.2 × 10^6^	-

## Supplementary Materials

The following are available online at https://www.mdpi.com/2073-4360/12/1/230/s1: Figure S1: The deconvoluted lines of XPS spectra: (A) the Br 3d core level of the linear polymer layer (sample SL3, Table 2), (B) the Br 3d core level of the star polymer layer (sample SG3, Table 2), and (C) the Br 3d core level of the benzophenone derivative layer (sample SBPH, Table 2), Figure S2: The deconvoluted lines of XPS spectra: (A) the C 1s core level after hydroxylation (sample SOH, Table 2), (B) the N 1s core level after hydroxylation (sample SOH, Table 2), (C) the C 1s core level after benzophenone derivative functionalization (sample SBPH, Table 2), and (D) the N 1s core level after benzophenone derivative functionalization (sample SBPH, Table 2), Table S1: Influence of polymer concentration in acetone/THF mixture used during spin-coating on layer thickness measured by ellipsometry, Figure S3: 1HNMR (600 MHz, D2O) of (A) linear PDMAEMA (sample L2, Table 1) and (B) linear QPDMAEMA (sample QL2), Figure S4: The dependence of fluorescein sodium salt absorbance on the concentration.
