# Supplementary material for: Nanolayers of Poly(N,N′-Dimethylaminoethyl Methacrylate) with a Star Topology and Their Antibacterial Activity
Source: Polymers (Basel). 2020 Jan 17;12(1):230. doi: 10.3390/polym12010230 (PMC7023597; doi:10.3390/polym12010230)
Supplement: Supplementary file 1 [file polymers-12-00230-s001.pdf]

Supplementary Materials

Nanolayers of Poly(*N*,*N'*-Dimethylaminoethyl Methacrylate) with a Star Topology and Their Antibacterial Activity

Paulina Teper [1], Joanna Chojniak-Gronek [2], Anna Hercog [1], Natalia Oleszko-Torbus [1], Grażyna Płaza [2], Jerzy Kubacki [3,4], Katarzyna Balin [3,4], Agnieszka Kowalczuk [1] and Barbara Mendrek [1,*]

1. Centre of Polymer and Carbon Materials, Polish Academy of Sciences, M. Curie-Sklodowskiej 34, 41-819 Zabrze, Poland; pteper@cmpw-pan.edu.pl (P.T.); ahercog@cmpw-pan.edu.pl (A.H.); noleszko@cmpw-pan.edu.pl (N.O.-T.); akowalczuk@cmpw-pan.edu.pl (A.K.)
2. Institute for Ecology of Industrial Areas, Kossutha 6, 40-844 Katowice, Poland; j.chojniak-gronek@ietu.pl (J.C.-G.); g.plaza@ietu.pl (G.P.)
3. A. Chelkowski Institute of Physics, University of Silesia, Uniwersytecka 4, 40-007 Katowice, Poland, jerzy.kubacki@us.edu.pl (J.K.); katarzyna.balin@us.edu.pl (K.B.)
4. Silesian Center for Education and Interdisciplinary Research, 75 Pulku Piechoty 1A, 41-500 Chorzów, Poland

* Correspondence: bmendrek@cmpw-pan.edu.pl; Tel.: +48-32-271-6077

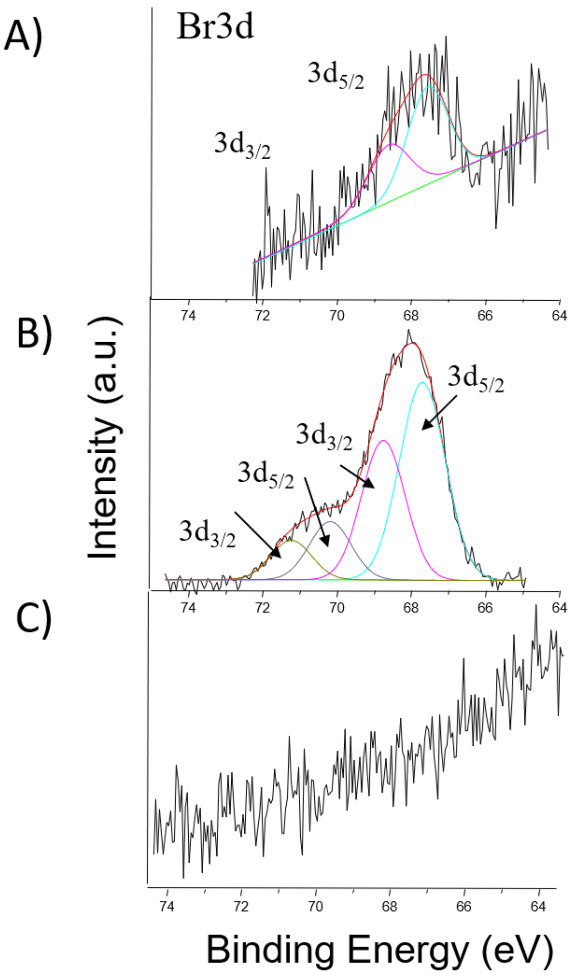

Figure S1. The deconvoluted lines of XPS spectra: A) the Br 3d core level of the linear polymer layer (sample SL3, Table 2), B) the Br 3d core level of the star

polymer layer (sample SG3, Table 2), C) the Br 3d core level of the benzophenone derivative layer (sample SBPH, Table 2).

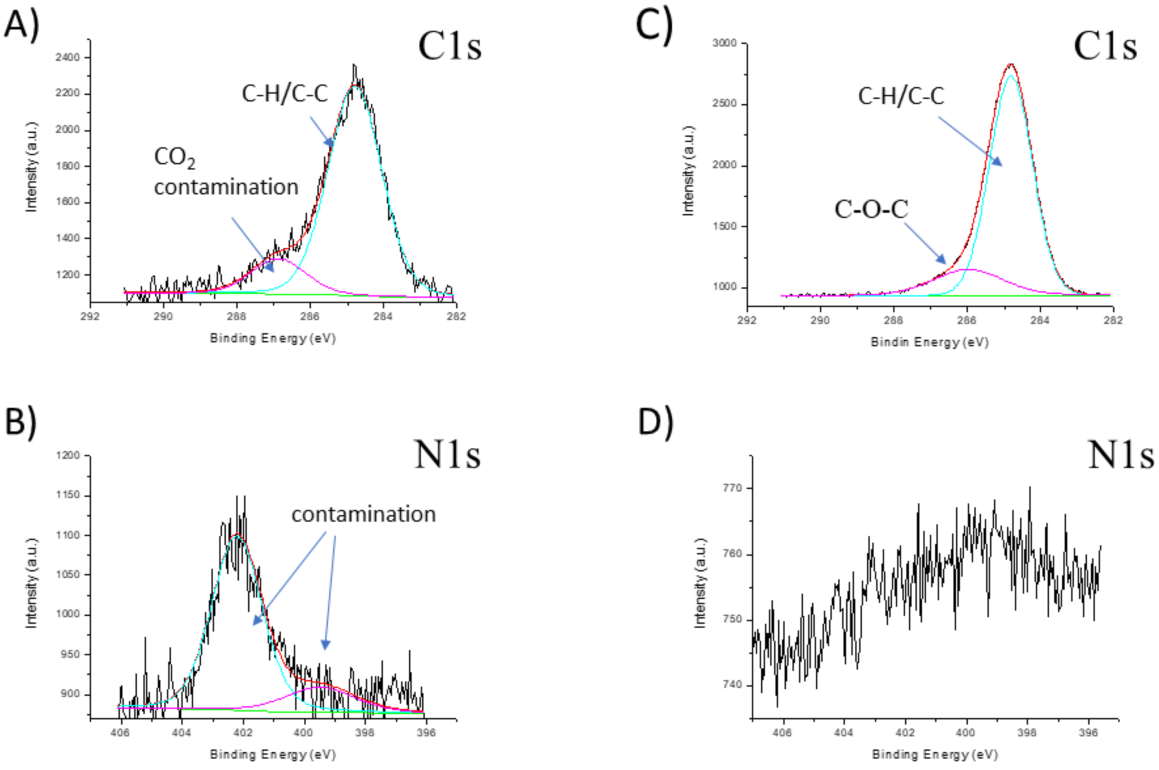

Figure S2. The deconvoluted lines of XPS spectra: A) the C 1s core level after hydroxylation (sample SOH, Table 2), B) the N 1s core level after hydroxylation (sample SOH, Table 2), C) the C 1s core level after benzophenone derivative functionalization (sample SBPH, Table 2), and D) the N 1s core level after benzophenone derivative functionalization (sample SBPH, Table 2).

Table S1. Influence of polymer concentration in acetone/THF mixture used during spin-coating on layer thickness measured by ellipsometry.

Sample	Concentration [wt%]	Thickness [nm]
L2	10	4.0
L2	20	2.2
L2	30	3.2
L2	46	2.9
L2	56	3.8
L2	>60	too viscous to form the layer
G2	1	70.0
G2	>1	too viscous to form the layer

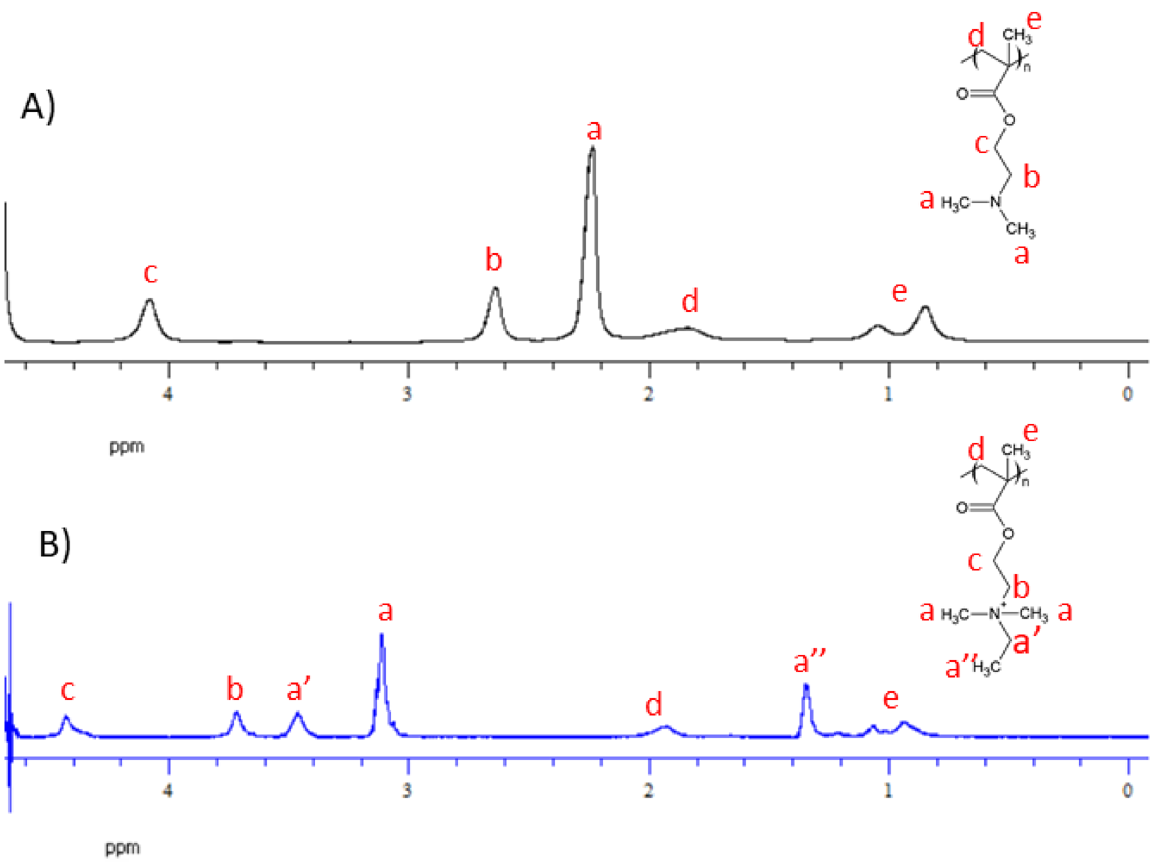

Figure S3. ^{1}HNMR (600 MHz, D_2O) of A) linear PDMAEMA (sample L2, Table 1), B) linear QPDMAEMA (sample QL2)

Sample SL2: δppm : 0.8-1.1 (CH_3C-), 1.7-2.0 (-CH_2C-), 2.2-2.3 (-NCH_3), 2.6-2.7 (-OCH_2CH_2N-) and 4.0-4.2 (-OCH_2CH_2N-)

Sample QSL2: δppm: 0.8-1.1 (CH_3C-), 1.3-1.4 (-N^+CH_2CH_2), 1.8-2.1 (-CH_2C-), 3.0-3.2 (-N^+CH_3), 3.4-3.5 (-N^+CH_2CH_2), 3.6-2.8 (-OCH_2CH_2N-) and 4.3-4.5 (-OCH_2CH_2N-)

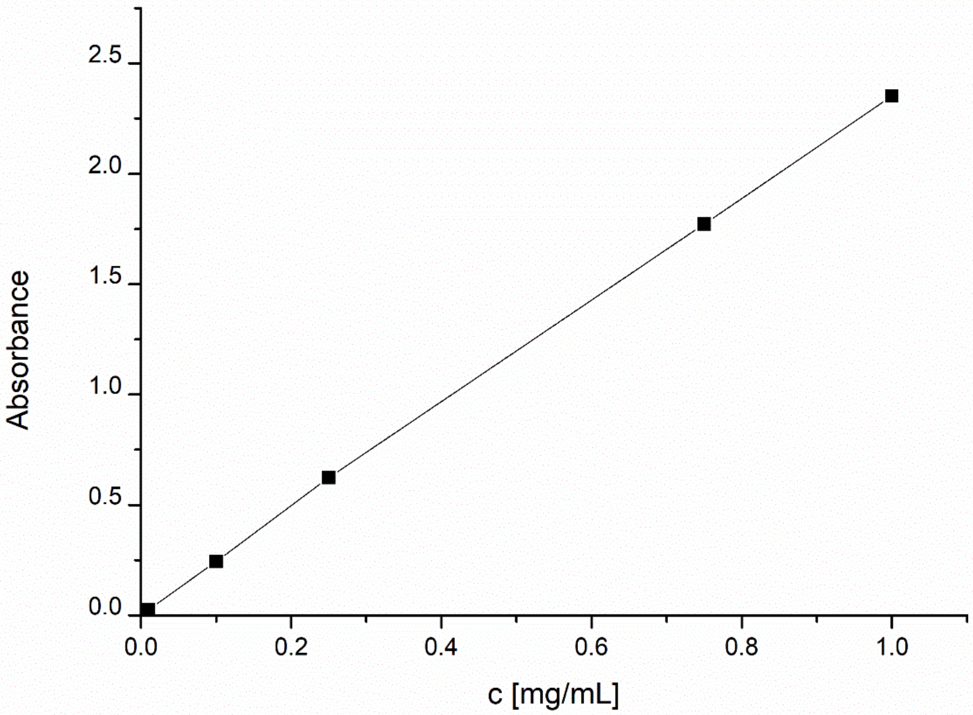

Figure S4. The dependence of fluorescein sodium salt absorbance on the concentration
